# Supplementary material for: Molecular genetic contributions to self-rated health
Source: Int J Epidemiol. 2016 Nov 13;46(3):994–1009. doi: 10.1093/ije/dyw219 (PMC5837683; doi:10.1093/ije/dyw219)
Supplement: Supplementary Materials [file supplementary_materials_dyw219.pdf]

Supplementary Materials for:

Harris et al. Molecular genetic contributions to self-rated health.

.

Contents

1. Supplementary Methods

Sources of genetic results from genome-wide association consortia

Polygenic profiling procedure

Sensitivity analysis

2. Supplementary Figures

Supplementary Figure 1: Regional plots showing the conditional analyses of the genome-wide significant regions with evidence of multiple signals.

Supplementary Figure 2: Enrichment analysis for general cognitive function using the 52 functional categories in the baseline model.

Supplementary Figure 3: Enrichment analysis for general cognitive function using the 10 cell-type specific categories in the baseline model.

3. Supplementary Tables

Supplementary Table 1: Details of the sources of genetic results from genome-wide association studies (GWAS) consortia.

Supplementary Table 2: BMI associated SNPs from Locke et al 2015 used in the Mendelian Randomisation.

Supplementary Table 3: Genome-wide significant SNP-based association results ($P < 5 \times 10^{-8}$)

Supplementary Tables 4-9: HLA allele analysis results

Supplementary Table 10: Genome-wide significant gene-based hits (P<2.8 x 10-6)

Supplementary Table 11: Functional annotation of the independent genome-wide significant SNPs.

Supplementary Table 12: DEPICT gene prioritisation results.

Supplementary Table 13: DEPICT gene set analysis results.

Supplementary Table 14: DEPICT tissue enrichment results.

Supplementary Table 15: Gene set analysis performed using MAGMA.

Supplementary Table 16: LD regression genetic correlations.

Supplementary Table 17: Number of SNPs included at each threshold for the polygenic profile scores.

Supplementary Table 18: Complete polygenic profile score associations with self-rated health using all five thresholds.

Sources of genetic results from genome-wide association consortia

CARDIoGRAM
Coronary artery disease data have been contributed by CARDIoGRAMplusC4D investigators.

CHARGE-Aging and Longevity
Longevity data have been provided by the CHARGE-Aging and Longevity consortium. Longevity was defined as reaching age 90 years or older. Genotyped participants who died between the ages of 55 and 80 years were used as the control group. There were 6036 participants who achieved longevity and 3757 participants in the control group across participating studies in the discovery meta-analysis.

Broer L, Buchman AS, Deelen J, Evans DS, Faul JD, Lunetta KL, Sebastiani P, Smith JA, Smith AV, Tanaka T, Yu L, Arnold AM, Aspelund T, Benjamin EJ, De Jager PL, Eirkisdottir G, Evans DA, Garcia ME, Hofman A, Kaplan RC, Kardia SL, Kiel DP, Oostra BA, Orwoll ES, Parimi N, Psaty BM, Rivadeneira F, Rotter JI, Seshadri S, Singleton A, Tiemeier H, Uitterlinden AG, Zhao W, Bandinelli S, Bennett DA, Ferrucci L, Gudnason V, Harris TB, Karasik D, Launer LJ, Perls TT, Slagboom PE, Tranah GJ, Weir DR, Newman AB, van Duijn CM and Murabito JM. **GWAS of Longevity in CHARGE Consortium Confirms APOE and FOXO3 Candidacy**. *J Gerontol A Biol Sci Med Sci*. 2015;70:110-8.

Acknowledgments
The CHARGE Aging and Longevity working group analysis of the longevity phenotype was funded through the individual contributing studies. The working group thanks all study participants and study staff**.**

CHARGE-Cognitive working group
General cognitive function data were obtained from the CHARGE Cognitive working group.

CHIC
Childhood cognitive ability data were obtained from the CHIC consortium.

DIAGRAM
Type 2 diabetes data were obtained from the DIAGRAM consortium.

Genetic Consortium for Anorexia nervosa
Anorexia nervosa data were obtained from the Genetic Consortium for Anorexia nervosa.

GIANT
BMI data were obtained from the GIANT consortium.

International Consortium for Blood Pressure (ICBP)
Blood pressure data were provided by ICBP.

International Genomics of Alzheimer's Project (IGAP)
Alzheimer's disease data were obtained from (IGAP)
Material and methods
International Genomics of Alzheimer's Project (IGAP) is a large two-stage study based upon genome-wide association studies (GWAS) on individuals of European ancestry. In stage 1, IGAP used genotyped and imputed data on 7 055 881 single nucleotide polymorphisms (SNPs) to meta-analyse four previously-published GWAS datasets consisting of 17 008 Alzheimer's disease cases and 37 154 controls (The European Alzheimer's disease Initiative – EADI the Alzheimer Disease Genetics Consortium – ADGC The Cohorts for Heart and Aging Research in Genomic Epidemiology consortium – CHARGE The Genetic and Environmental Risk in AD consortium – GERAD). In stage 2, 11 632 SNPs were genotyped and tested for association in an independent set of 8572 Alzheimer's

disease cases and 11 312 controls. Finally, a meta-analysis was performed combining results from stages 1 & 2. Only stage 1 data were used for LD Score regression and polygenic risk score analyses.

Acknowledgments
We thank the International Genomics of Alzheimer's Project (IGAP) for providing summary results data for these analyses. The investigators within IGAP contributed to the design and implementation of IGAP and/or provided data but did not participate in analysis or writing of this report. IGAP was made possible by the generous participation of the control subjects, the patients, and their families. The i–Select chips was funded by the French National Foundation on Alzheimer's disease and related disorders. EADI was supported by the LABEX (laboratory of excellence program investment for the future) DISTALZ grant, Inserm, Institut Pasteur de Lille, Université de Lille 2 and the Lille University Hospital. GERAD was supported by the Medical Research Council (Grant n° 503480), Alzheimer's Research UK (Grant n° 503176), the Wellcome Trust (Grant n° 082604/2/07/Z) and German Federal Ministry of Education and Research (BMBF): Competence Network Dementia (CND) grant n° 01GI0102, 01GI0711, 01GI0420. CHARGE was partly supported by the NIH/NIA grant R01 AG033193 and the NIA AG081220 and AGES contract N01–AG–12100, the NHLBI grant R01 HL105756, the Icelandic Heart Association, and the Erasmus Medical Center and Erasmus University. ADGC was supported by the NIH/NIA grants: U01 AG032984, U24 AG021886, U01 AG016976, and the Alzheimer's Association grant ADGC–10–196728.

METASTROKE
Ischaemic stroke data were obtained from the METASTROKE consortium. The METASTROKE consortium is supported by NINDS (NS017950). We thank all study participants, volunteers, and study personnel that made this consortium possible. The METASTROKE study consists of combined data from 15 GWAS of IS (12 389 cases vs 62 004 controls). We used TOAST criteria17 to classify IS as large artery stroke (LAS) (2167 cases/49 159 controls from 11 studies), cardioembolic stroke (CE) (2365 cases/ 56,140 controls from 13 studies), and small vessel disease (SVD) (1894 cases/51 976 controls from 12 studies). METASTROKE studies consisted of independently performed genome-wide single nucleotide polymorphism (SNP) genotyping using standard technologies and imputation to HapMap release 21 or 22 CEU phased genotype18 or 1000 Genome reference panels. Investigators contributed summary statistical data from association analyses using frequentist additive models for metaanalysis after application of appropriate quality control measures. Polygenic scores reveal combined effects of multiple nonsignificant variants derived from a derivation sample and tested in an independent replication sample. We derived polygenic scores for multiple p value cutoffs (0·5, 0·25, 0·1, 0·05, 0·01, 0·001, and 0·0001) in derivation samples.

Psychiatric Genetics Consortium
Schizophrenia, bipolar disorder, major depressive disorder and ADHD data were obtained from the Psychiatric Genetics Consortium.

Social Science Genetic Association Consortium
Years of education data were obtained from the Social Science Genetic Association Consortium.

SpiroMeta/CHARGE Pulmonary
Lung function data were obtained from the SpiroMeta and CHARGE Pulmonary group.

The Genetics of Personality Consortium
Neuroticism data were obtained from the Genetics of Personality consortium.

Polygenic profiling procedure

The genetic data files (.map and .ped files) supplied from Biota (the UK Biobank online repository) were unsuitable for use in the polygenic profile analyses as the .ped allele coding used a 1, 2 numeric allele encode rather than the standard ACGT encode format. In order to enable the analysis, the .ped files were recoded to the standard encode format. To achieve this, a bespoke programme was developed to create new files using a lookup-substitution method. A fast-in-memory lookup string hash table was created to hold the SNP-ID, along with the allele identifiers for the SNP. A simple loop then performed serialised lookups based on string position, to create an associated string with the correct ACGT encode. This was then appended to the six mandatory data fields extracted from initial string. In order to maximise performance and enable timely completion of the lookup-substitution, these loops were run in parallel threads in a standard multiprocessor environment.

Sensitivity analysis

To test whether FDR significant associations between polygenic risk for coronary artery disease, and Verbal-numerical reasoning and educational attainment were confounded by individuals diagnosed with cardiovascular disease, 2779 individuals who had had a heart attack and 2521 individuals with angina were removed from the regression analysis. Similarly 5800 individuals with diabetes (type 1 or type 2) were removed from the regression investigating an association between polygenic risk for type 2 diabetes and educational attainment. Finally, 26 912 individuals with hypertension were removed from the regression analysis investigating the association between polygenic risk for systolic blood pressure and educational attainment.

Supplementary Figure 1. Regional plots showing the conditional analyses of the genome-wide significant regions with evidence of multiple signals. Analyses were conditioned on the most significant SNP in each region. The circles represent individual SNPs, with the colour indicating pairwise linkage disequilibrium (LD) to the most significant SNP from the original analysis (calculated from 1000 Genomes Nov 2014 EUR). The solid blue line indicates the recombination rate and –log10 P-values are shown on the y-axis.

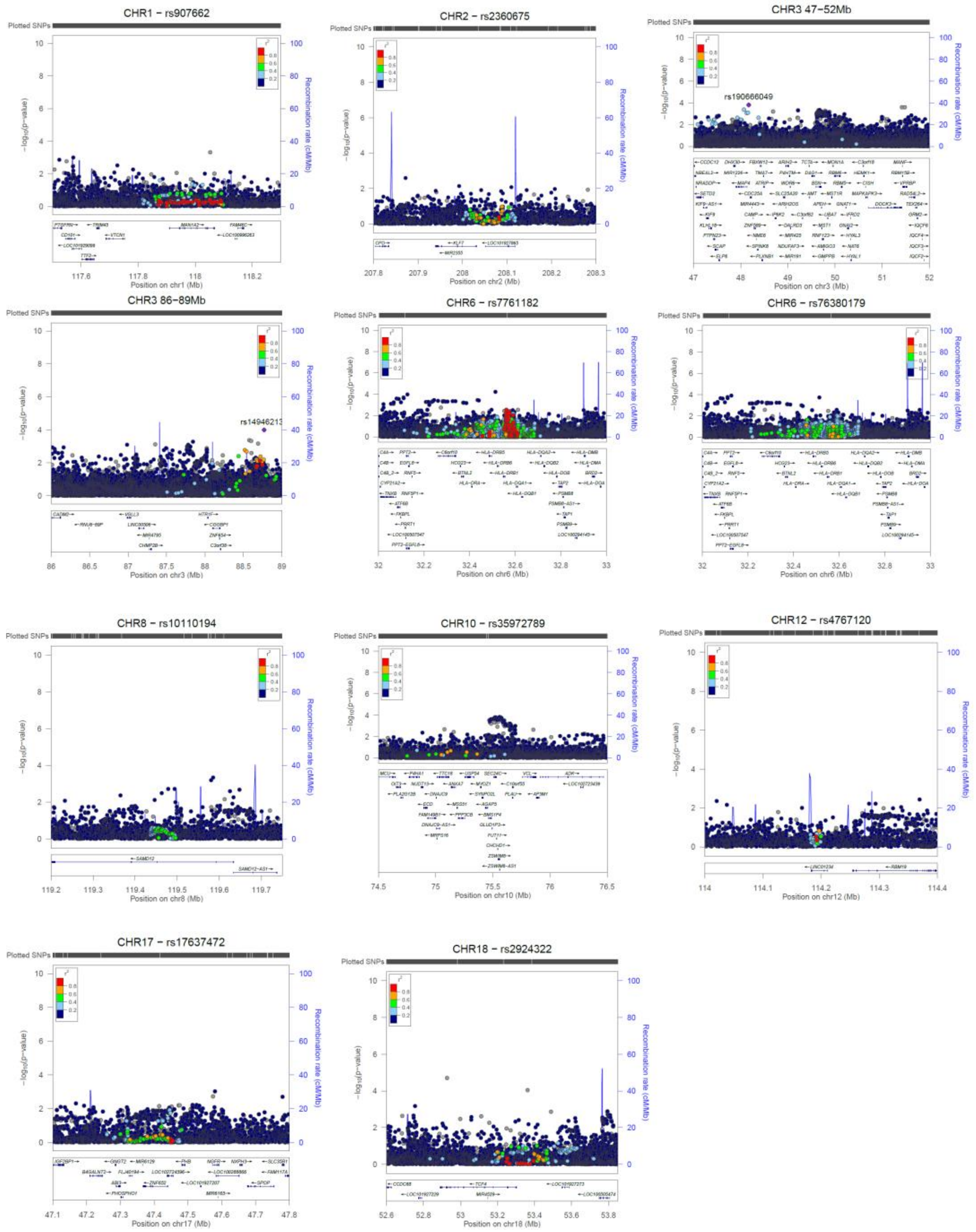

Supplementary Figure 2. Enrichment analysis for general cognitive function using the 52 functional categories in the baseline model. The enrichment statistic is the proportion of heritability found in each functional group divided by the proportion of SNPs in each group ($Pr(h^2)/Pr(SNPs)$). The dashed line indicates no enrichment found when $Pr(h^2)/Pr(SNPs) = 1$. Statistical significance is indicated by asterisk. FDR correction indicates significance at 0.00638.

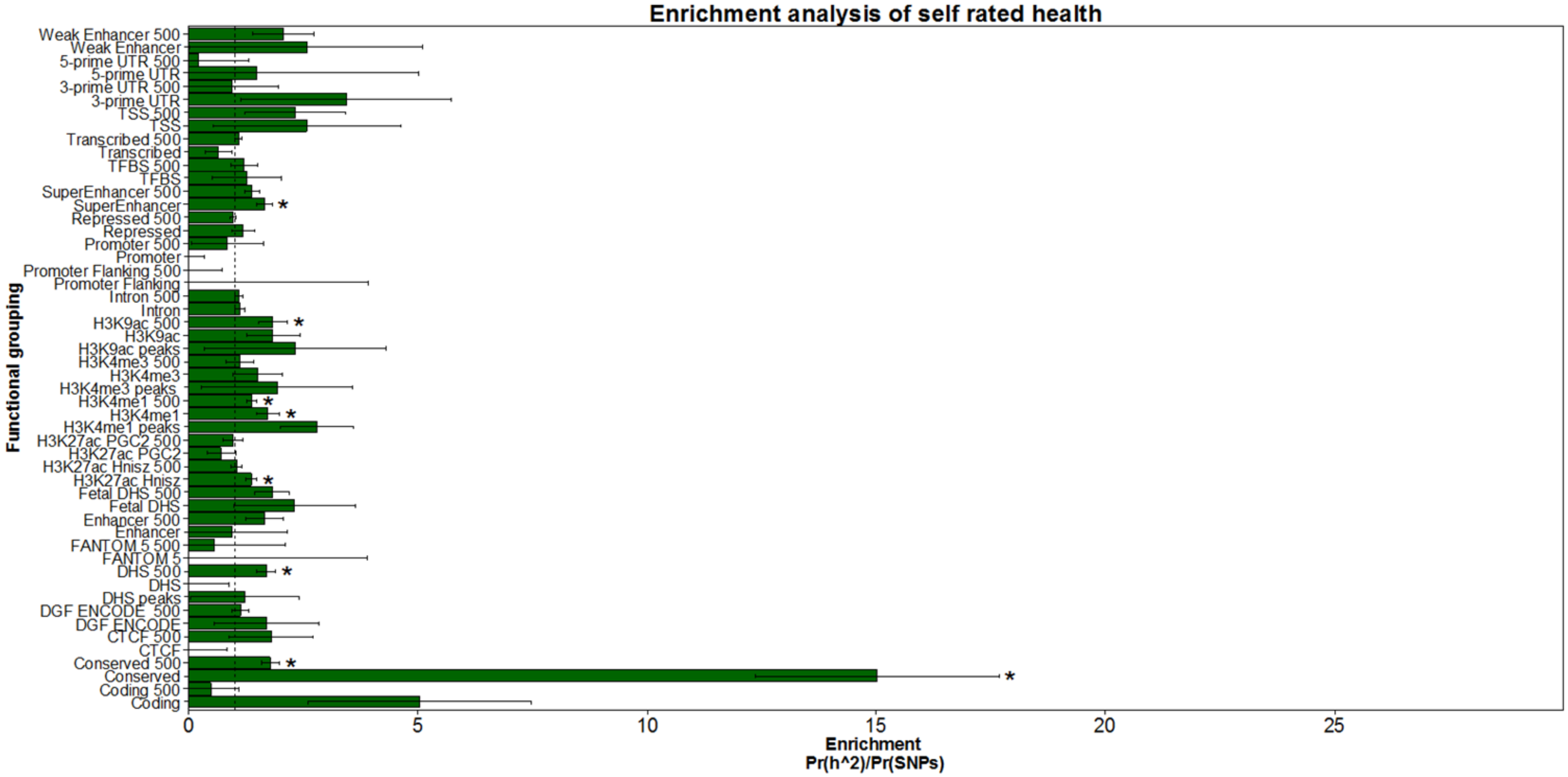

Supplementary Figure 3. Enrichment analysis for general cognitive function using the 10 cell-type specific categories in the baseline model. The enrichment statistic is the proportion of heritability found in each functional group divided by the proportion of SNPs in each group ($Pr(h^2)/Pr(SNPs)$). The dashed line indicates no enrichment found when $Pr(h^2)/Pr(SNPs) = 1$. Statistical significance is indicated by asterisk. FDR correction indicates significance at 1.68x10-7

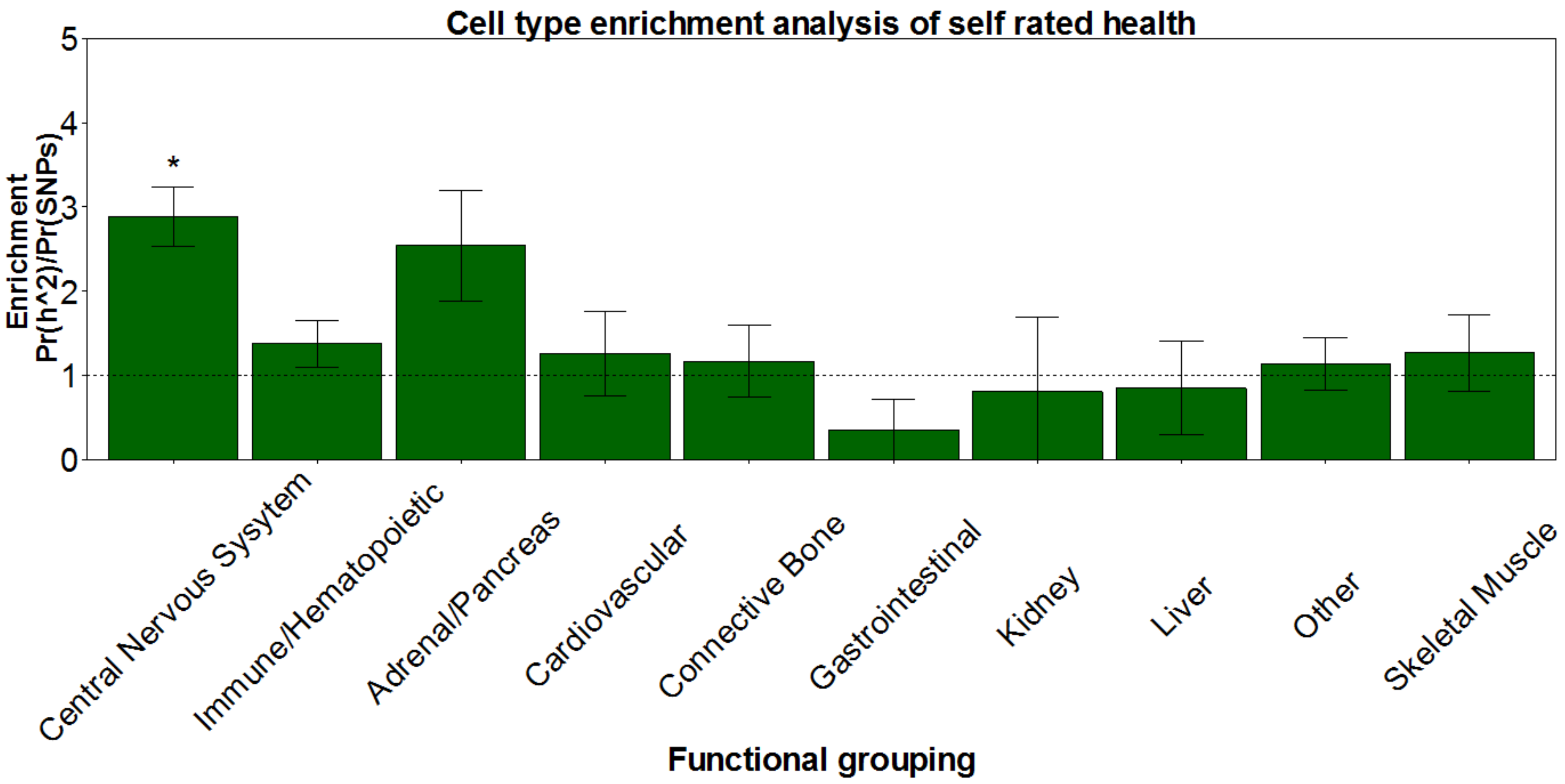

Supplementary Table 1
Sources of genetic results from genome-wide association consortia

Phenotype	Consortium	URL	Reference	No. of individuals in GWAS
Years of Education	Social Science Genetic Association Consortium	http://ssgac.org/Data.php	Rietveld et al. Science 2013; 314: 1467-1471. PMID: 23722424	101 069
Childhood cognitive ability	CHIC	http://ssgac.org/Data.php	Benyamin et al. Mol Psychiatr 2014; 19: 253-258. PMID: 23358156	17 989
General Cognitive Function	CHARGE Cognitive working group		Davies et al. Mol Psychiatr 2015; 20: 183-192 PIMD: 25644384	53 949
Neuroticism	The Genetics of Personality Consortium	http://www.tweelingenregister.org/GPC/	De Moor, et al. JAMA Psychiatry 2015; 72(7):642-650. PMID: 25993607	63 661
BMI	GIANT	http://www.broadinstitute.org/collaboration/ giant/index.php/GIANT_consortium_data_files	Locke et al. Nature 2015; 518: 197-206. PMID: 25673413	339 224
Longevity	CHARGE-Aging and Longevity working group		Broer et al. J Gerontol A Biol Sci Med Sci 2015; 70: 110-118. PMID: 25199915	6036 cases<br>3757 controls
ADHD	Psychiatric Genetics Consortium (PGC)	https://www.med.unc.edu/pgc/downloads	Cross-Disorder Group of the Psychiatric Genomics Consortium. Lancet 2013; 381: 1371-1379. PMID: 23453885	1947 trio cases 1947 trio pseudocontrols,

				840 cases<br>688 controls
Alzheimer's disease	International Genomics of Alzheimer's Project (IGAP)	http://www.pasteur-lille.fr/en/recherche/u744/igap/igap_download.php	Lambert et al. Nat Genet 2013; 45: 1452-1458. PMID: 24162737	17 008 cases<br>37 154 controls
Anorexia nervosa	Genetic Consortium for Anorexia Nervosa (GCAN)	http://www.med.unc.edu/pgc/downloads	Boraska, Vesna, et al. Molecular psychiatry 2014; 19(10): 1085-1094. PIMD: 24514567	2 907 cases 14 860 controls
Bipolar disorder	Psychiatric Genetics Consortium (PGC)	https://www.med.unc.edu/pgc/downloads	Psychiatric GWAS Consortium Bipolar Disorder Working Group. Nat Genet 2011; 43: 977-983. PMID: 21926972	7481 cases<br>9250 controls
Major depressive disorder	Psychiatric Genetics Consortium (PGC)	https://www.med.unc.edu/pgc/downloads	Major Depressive Disorder Working Group of the Psychiatric GWAS Consortium. Mol Psychiatr 2013; 18: 497-511. PMID: 22472876	9240 cases<br>9519 controls
Schizophrenia	Psychiatric Genetics Consortium (PGC)	https://www.med.unc.edu/pgc/downloads	Schizophrenia Working Group of the Psychiatric Genomics Consortium. Nature 2014; 511: 421-427. PMID: 25056061	36 989 cases<br>113 075 controls
Forced expiratory volume in 1 second (FEV_1)	SpiroMeta/CHARGE-Pulmonary		Soler Artigas et al. Nature Genetics 2011; 43: 1082-1090. PMID: 21946350	48 201
Blood pressure:	International Consortium of Blood		Ehret et al. (2011) Nature 478, 103-109.	69 395

Diastolic	Pressure (ICBP)		PMID: 21909115	
Blood pressure: Systolic	International Consortium of Blood Pressure (ICBP)		Ehret et al. Nature 2011; 478: 103-109. PMID: 21909115	69 395
Coronary Artery Disease	CARDIoGRAM	http://www.cardiogramplusc4d.org/downloads/	Schunkert et al. Nat Genet 2011; 43: 333-338. PMID: 21378990	22 233 cases<br>64 762 controls
Stroke: Ischaemic	METASTROKE	http://www.strokegenetics.com/members-area/meta-stroke	Traylor et al. Lancet Neurol 2012; 11: 951-962. PMID: 23041239	12 389 cases<br>62 004 controls
Stroke: Cardioembolic	METASTROKE	http://www.strokegenetics.com/members-area/meta-stroke	Traylor et al. Lancet Neurol 2012; 11: 951-962. PMID: 23041239	2365 cases<br>62 004 controls
Stroke: Large vessel disease	METASTROKE	http://www.strokegenetics.com/members-area/meta-stroke	Traylor et al. Lancet Neurol 2012; 11: 951-962. PMID: 23041239	2167 cases<br>62 004 controls
Stroke: Small vessel disease	METASTROKE	http://www.strokegenetics.com/members-area/meta-stroke	Traylor et al. Lancet Neurol 2012; 11: 951-962. PMID: 23041239	1894 cases<br>62 004 controls
Type 2 diabetes	DIAGRAM	http://diagram-consortium.org/downloads.html	Morris et al. Nat Genet 2012; 44: 981-990. PMID: 22885922	12 171 cases<br>56 862 controls

Supplementary Table 2. BMI associated SNPs from Locke et al 2015 used in the Mendelian Randomisation.
(See attached Excel spreadsheet)

Supplementary Table 3. Genome-wide significant SNP-based association results for self-rated health (P<5 x 10-8) (excel file).The results are ordered by significance of the association. The independent SNP signals, as identified by the LD Clumping analysis, are highlighted in red.
(See attached Excel spreadsheet)

Supplementary Tables 4-9. HLA allele analysis results.
(See attached Excel spreadsheet)

Supplementary Table 10. Genome-wide significant gene-based hits (P<2.8 x 10-6) in the MAGMA gene-based analysis. NSNPS is the number of SNPs in the gene; Effect Size is the number of independent SNPs in the gene.
(See attached Excel spreadsheet)

Supplementary Table 11. Functional annotation of the independent genome-wide significant SNPs. All information contained in this table was extracted from the GTEx database (http://www.broadinstitute.org/gtex/) and the Regulome DB database (http://regulome.stanford.edu/index).
(See attached Excel spreadsheet)

Supplementary Table 12. Gene prioritization for self-rated health. Locus shows the rs numbers for the SNPs in an associated region. N genes indicates the number of genes found within each locus. Gene closest to lead SNP states if the gene prioritised is closest to the most associated SNP in the region. Top cis eQTL SNP provides the rs number for SNPs that are known to be eQTLs based on whole blood.
(See attached Excel spreadsheet)

Supplementary Table 13. Gene set analysis for self-rated health. Reconstituted gene set Z scores indicates the strength of the association between a given gene and the reconstituted gene set.
(See attached Excel spreadsheet)

Supplementary Table 14. Results of tissue enrichment for self-rated health. Mesh pertains to the medical subject heading (http://www.nlm.nih.gov/mesh/). Tissue specific expression Z score indicates which genes were highly expressed in the tissue that overlap with the associated loci. The z score indicates the level of tissue specific expression.
(See attached Excel spreadsheet)

Supplementary Table 15. Gene set analysis performed using MAGMA. NGENES, number of genes in a gene set; SELF P, p value derived using self-contained testing; COMP P, p value derived through competitive testing. FDR correction indicates that there were no significantly enriched gene-sets.
(See attached Excel spreadsheet)

Supplementary Table 16. Genetic correlations between self-rated health in UK Biobank and the health-related variables collected from GWAS consortia. Statistically significant p-values (after False Discovery Rate correction; threshold: $p < 0.0061$) are shown in bold. There was no evidence for a sufficient polygenic signal in the small vessel disease data set and so no genetic correlation could be derived.
(See attached Excel spreadsheet)

Supplementary Table 17. Number of SNPs included at each threshold for the polygenic profile scores.
(See attached Excel spreadsheet)

Supplementary Table 18. Associations between polygenic profiles of health related traits, and self-rated health controlling for age, sex, assessment centre, genotyping batch and array, and ten principal components for population structure. Statistically significant values (P<0.024) are shown in bold. Self-rated health is scored such that higher scores indicate better rated health.
(See attached Excel spreadsheet)
